# Triglyceride Glucose-Waist Circumference (TyG-WC) Is a Reliable Marker to Predict Non-Alcoholic Fatty Liver Disease

**DOI:** 10.3390/biomedicines10092251

**Published:** 2022-09-11

**Authors:** Seungah Song, Da-Hye Son, Su-Jung Baik, Wan-Je Cho, Yong-Jae Lee

**Affiliations:** 1 Department of Family Medicine, Yonsei University College of Medicine, Seoul 06273, Korea; 2 Healthcare Research Team, Health Promotion Center, Gangnam Severance Hospital, Seoul 06273, Korea

**Keywords:** NAFLD, TyG index, TyG-WC, TyG-BMI, fatty liver index, insulin resistance, prediction, diagnosis, body composition, Korean

## Abstract

The triglyceride and glucose index (TyG index), a marker of insulin resistance, is positively associated with NAFLD. Modified TyG indices, combining body composition markers including body-mass index (BMI) or waist circumference (WC) with the TyG index, are reported to enhance predictability of insulin resistance. This study aimed to compare the usefulness of modified TyG indices for predicting NAFLD with the TyG index and fatty liver index (FLI). This cross-sectional study included 12,757 Korean adults. The TyG index and FLI were calculated using established formulas, and TyG-BMI and TyG-WC were calculated as TyG × BMI and TyG × WC, respectively. All measures were divided into quartiles. NAFLD severity (grade 0–3) was compared using ANOVA by quartiles of each index. Odds ratios (ORs) and 95% confidence intervals (CIs) for NAFLD were calculated using a multiple logistic regression analysis. ROC and AUROC analyses were performed to compare the predictability of NAFLD using WC, BMI, TyG, TyG-BMI, TyG-WC, and FLI. A higher TyG index, TyG-BMI, TyG-WC, and FLI were associated with a higher grade of NAFLD. ORs (CIs) for NAFLD increased in all indices, especially in TyG-WC (39.251 (31.304–49.215)) and FLI (38.937 (31.145–48.678)). AUROC was 0.848 (0.840–0.855) for TyG-WC and 0.850 (0.842–0.857) for FLI. TyG-WC is a reliable indicator for the presence of NAFLD in Korean adults.

## 1. Introduction

Non-alcoholic fatty liver disease (NAFLD) is characterized by the diffuse accumulation of triglycerides in hepatocytes not caused by excessive alcohol intake and other causes of liver disease. NAFLD is generally attributed to obesity-induced insulin resistance [1]. With the growing epidemic of obesity, NAFLD is one of the most prevalent causes of chronic liver disease, ranging from 25% to 45% [2]. In Korea, the prevalence of NAFLD was between 10 and 15% in non-obese and between 55 and 70% in obese individuals [3,4]. NAFLD encompasses a spectrum of clinical syndromes, ranging from simple steatosis to non-alcoholic steatohepatitis that may progress to advanced fibrosis, cirrhosis, and cirrhosis complicated by hepatocellular carcinoma [5,6,7,8]. NAFLD is clinically important because of its association with an increased risk of type 2 diabetes, cardiovascular disease, and chronic kidney disease, as well as liver-related diseases [7,9,10,11]. Thus, early identification of individuals at higher risk for NAFLD may allow for the application of preventive strategies that can slow the morbidity and mortality of both liver-related and cardiometabolic diseases.

The traditional diagnosis of NAFLD requires various techniques such as liver ultrasonography, magnetic resonance, and biopsy [4]; however, these procedures are either invasive or expensive, with limited applicability in general clinical practice. The most widely used diagnostic tool is liver ultrasonography, but this, too, shows operator-dependence. The general hypothesis is that in NAFLD, an excess of visceral adipose tissue affects glucose metabolism and insulin resistance. Because of this, the fatty liver index (FLI), calculated using body mass index (BMI), waist circumference (WC), triglycerides (TG), and γ-glutamyltransferase (GGT), has emerged as a simple and economical alternative tool for mass screening for hepatic steatosis with reasonable sensitivity and specificity [12]. In addition, a laboratory marker of insulin resistance, such as the triglyceride and glucose index (TyG index), has been positively associated with the presence of NAFLD [7,13,14]. More recently, several researchers have shown that the modified TyG index, which combines the TyG index with the effect of body composition including BMI or WC, enhances predictability of insulin resistance [15,16]. However, no large-scale studies have investigated the relationship between the modified TyG index and the presence of NAFLD. Therefore, this study aimed to compare the usefulness of the modified TyG index to that of the TyG index and FLI in predicting NAFLD.

## 2. Materials and Methods

### 2.1. Study Participants

**Figure 1:**
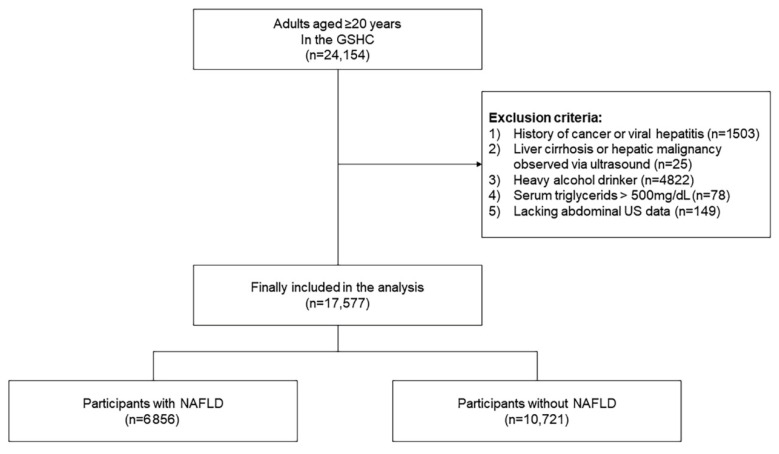
Study flow chart.

This study was conducted using data from regular health examinations at the Health Promotion Center, Gangnam Severance Hospital, Yonsei University College of Medicine, between January 2017 and October 2020. Out of 27,554 participants enrolled in the survey during the study period, data from 24,154 Koreans aged 20 years and older were included. Of these individuals, we excluded those meeting at least one of following criteria (*n* = 6577): those without abdominal sonography results (*n* = 149); those with a history of cancer or viral hepatitis (*n* = 1503); those with liver cirrhosis observed via ultrasound (*n* = 25); those with triglycerides > 500 mg/dL (*n* = 78); and heavy alcohol drinkers (*n* = 4822). Following these exclusions, data from 17,577 participants were included in the final analysis (Figure 1). Written informed consent was obtained from each patient included in the study. This study was conducted in accordance with the ethical principles of the Declaration of Helsinki and was approved by the Institutional Review Board of Yonsei University College of Medicine, Seoul, Korea (IRB number: 3-2021-0093).

### 2.2. Data Collection

The social and medical history of each participant was obtained by a self-administered questionnaire that included questions regarding smoking, alcohol intake, physical activity, medications, and history of other diseases. Smoking status was categorized as non-smoker, ex-smoker, or current smoker. Excessive alcohol drinking was defined as consumption at least twice a week. Regular exercise was defined as exercise at least three times a week. Weight and height were measured to the nearest 0.1 kg and 0.1 cm, respectively, in light indoor clothing without shoes. Body mass index (BMI) was calculated as the weight in kilograms divided by the square of the height in meters (kg/m^2^). Systolic blood pressure (SBP) and diastolic blood pressure (DBP) were measured using the patient’s right arm and a standard mercury sphygmomanometer (Baumanometer, W.A. Baum Co., Inc., Copiague, NY, USA) after five minutes of rest. All blood samples were obtained from the antecubital vein after a 12-h overnight fast. Fasting plasma glucose, triglycerides, high-density lipoprotein cholesterol (HDL-C), low-density lipoprotein cholesterol (LDL-C), aspartate aminotransferase (AST), alanine aminotransferase (ALT), and GGT levels were measured by enzymatic methods using a chemistry analyzer (Hitachi 7600, Hitachi Co., Tokyo, Japan). Hypertension was defined as SBP ≥ 140 mmHg, DBP ≥ 90 mmHg, or current use of hypertension medication. Type 2 diabetes was defined by a fasting plasma glucose level ≥ 126 mg/dL or current use of anti-diabetic agents or insulin. Dyslipidemia was defined as triglyceride ≥ 150 mg/dL, low HDL-cholesterol < 50 mg/dL, or current use of dyslipidemia medications. Non-obese was defined as BMI under 25 kg/m^2^ according to the obesity classification of the Asia-Pacific guidelines [17]. Based on the International Diabetes Federation (IDF), metabolic syndrome was defined as individuals who have central adiposity plus two or more of the followings: (1) fasting glucose ≥100 mg/dL or previously diagnosed type 2 diabetes; (2) serum TG ≥ 150 mg/dL or treatment with lipid-lowering agents; (3) serum HDL-C < 40 mg/dL for men and <50 mg/dL for women; (4) SBP > 130 mHg or DBP > 85 mmHg or treatment with anti-hypertensive agents. Viral hepatitis was defined as positive results for the hepatitis B surface antigen or anti-hepatitis C virus antibody. The levels of hepatitis B surface antigen and anti-hepatitis C virus antibodies were measured using a Roche E-170 device (Roche Diagnostics, Mannheim, Germany).

### 2.3. FLI, TyG Index and TyG Related Parameters

The FLI was computed using the formula [12]:

FLI = (e ^0.953 × loge (TG) + 0.139 × BMI + 0.718 × loge (GGT) + 0.053 × WC − 15.745)^/(1 + e ^0.953 × loge (TG) + 0.139 × BMI + 0.718 × loge (GGT) + 0.053 × WC − 15.745)^ × 100

The TyG index was calculated using the formula [13]:

Tyg Index = ln [fasting TGs (mg/dL) × fasting glucose (mg/dL)/2]

TyG-WC and TyG-BMI are defined as TyG index × WC and TyG index × BMI, respectively [14].

### 2.4. NAFLD

Fatty liver disease was diagnosed based on the findings of an abdominal ultrasonography scan with a 3.5-MHz transducer (HDI 5000, Philips, Bothell, WA, USA). Ultrasonography was performed by two experienced radiologists who were unaware of the aims of the study and blinded to laboratory findings; the coefficients of variations for inter- and intra-operator reproducibility were 6.8% and 4.3%, respectively. Livers with any degree of hepatic steatosis were considered as having NAFLD in the present study. The severity of NAFLD was classified into four grades: grade 0 (absence of steatosis with normal echogenicity), grade 1 (slight diffuse increase in bright homogeneous echoes in the liver parenchyma, with normal visualization of the diaphragm and portal and hepatic vein borders and normal hepatorenal contrast), grade 2 (moderate steatosis, impaired echogenicity of the main portal vein wall), and grade 3 (marked increase in bright echoes at a shallow depth, with deep attenuation and impaired visualization of the diaphragm and marked vascular blurring) [18]. 

### 2.5. Statistical Analysis

Clinical characteristics of the study population were compared using an independent two sample t-test; chi-square tests were used for categorical variables. Fatty liver severity was compared using a one-way analysis of variance (ANOVA) test according to the quartile of each index. The odds ratios (ORs) and 95% confidence intervals (CIs) for NAFLD were calculated using a multiple logistic regression analysis after adjusting for confounding variables, including AST, ALT, SBP, DBP, presence of type 2 diabetes, smoking status, and exercise status, across TyG and TyG-related parameter quartiles. Receiver operating characteristic (ROC) and area under the ROC curve (AUROC) analyses were performed without adjustment to compare the predictability of NAFLD between WC, BMI, TyG, TyG-BMI, TyG-WC, and FLI. The comparison of ROCs was performed using the Delong method. All analyses were conducted using the SPSS software (version 25; IBM Corp., Armonk, NY, USA). All statistical tests were two-tailed, and statistical significance was determined at *p* < 0.05. 

## 3. Results

A total of 17,577 participants were enrolled in this study. Table 1 presents the clinical characteristics of the study population according to NAFLD status. There were 6856 participants with NAFLD, among whom 4127 (60.2%) were male. The mean age in the group with NAFLD was 51.1 ± 11.8 years, and the group without NAFLD had a mean age of 47.5 ± 13.2 years. The mean age, BMI, WC, AST, ALT, fasting glucose, GGT, total cholesterol, HDL-C, LDL-C, and triglycerides were significantly higher in the study population with NAFLD than in the group with normal liver sonography. The proportion of smokers, hypertension, type 2 diabetes, and metabolic syndrome were also higher in the study population with NAFLD.

**Figure 2:**
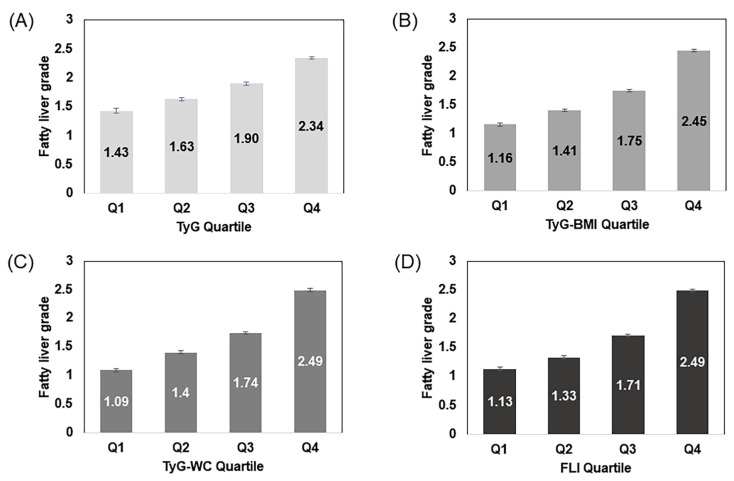
Fatty liver severity according to quartiles of each parameter. TyG, triglyceride and glucose; TyG-BMI, TyG-body mass index; TyG-WC, TyG-waist circumference; FLI, fatty liver index. (**A**) Mean values of fatty liver grade according to TyG quartiles; (**B**) Mean values of fatty liver grade according to TyG-BMI quartiles; (**C**) Mean values of fatty liver grade according to TyG-WC quartiles; (**D**) Mean values of fatty liver grade according to FLI quartiles.

Figure 2 shows the fatty liver severity according to quartiles of TyG index, TyG-BMI, TyG-WC, and FLI. The higher the indices, the higher was the fatty liver grade for all indices. The increase in the fatty liver severity was more pronounced in the TyG-WC index and FLI measurements. 

The ORs (95% CIs) for NAFLD according to the quartiles of each parameter are shown in Table 2. Compared to the lowest TyG quartile in all participants, the OR (95% CI) for NAFLD was 8.656 (7.633–9.817) in the highest quartile after adjusting for age, AST, ALT, SBP, DBP, smoking, exercise, hypertension, and type 2 diabetes. The TyG-BMI index OR (95% CI) for NAFLD was 26.815 (22.884–31.422), the TyG-WC index OR (CI) was 34.515 (28.716–47.485) for the TyG-WC index, and the FLI OR (CI) was 34.564 (28.831–41.437). When stratified by gender, the ORs (95% CIs) for NAFLD in male participants were 5.602 (4.761–6.592) for the TyG index, 10.435 (8.752–12.442) for TyG-BMI, 12.912 (10.592–15.740) for TyG-WC, and 12.299 (10.018–15.099) for FLI. Similar results were observed in female participants: 9.381 (7.722–11.396) for the TyG index, 25.194 (19.902–31.893) for TyG-BMI, 32.163 (24.546–42.143) for TyG-WC, and 31.367 (24.054–40.903) for FLI.

**Figure 3:**
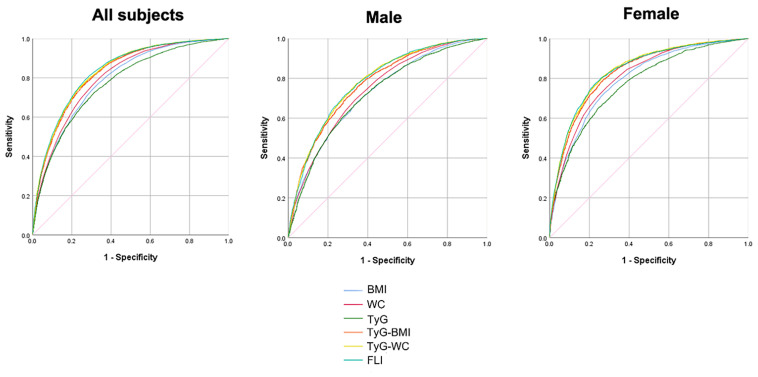
Receiver operating characteristic (ROC) curves of each parameter for predicting NAFLD. BMI, body mass index; WC, waist circumference; TyG, triglyceride and glucose; FLI, fatty liver index.

Figure 3 and Table 3 illustrate the predictability of NAFLD, illustrated by areas under the ROC curves, among WC, BMI, TyG, TyG-BMI, TyG-WC, and FLI. The AUROC was 0.790 (0.783–0.797) for BMI, 0.803 (0.796–0.810) for WC, 0.773 (0.765–0.780) for the TyG index, 0.827 (0.821–0.834) for TyG-BMI, 0.832 (0.826–0.839) for TyG-WC, and 0.835 (0.828–0.841) for FLI. The AUROC of TyG-WC and FLI were not statistically different, with the post-hoc *p*-value of 0.132, and TyG-WC and FLI showed better predictability for NAFLD compared to other indices. 

Table 4 shows the predictability of NAFLD of modified TyG indices according to the presence of metabolic syndrome, type 2 diabetes, and obesity. TyG-WC showed better predictability in non-obese individuals without metabolic syndrome or type 2 diabetes. The AUROC for TyG-WC was 0.804 (0.795–0.814) in the non-obese group, 0.716 (0.701–0.731) in the obese group, 0.831 (0.824–0.838) in individuals without type 2 diabetes, 0.716 (0.683–0.748) in individuals with type 2 diabetes, 0.812 (0.804–0.820) in individuals without metabolic syndrome, and 0.669 (0.644–0.693) in individuals with metabolic syndrome. 

## 4. Discussion

In this cross-sectional study, different parameters were tested to predict the presence of NAFLD. A greater value of TyG-WC and FLI was associated with a higher grade of NAFLD. When divided into quartiles, the highest quartiles of TyG-WC and FLI showed greater ORs for NAFLD compared to the lowest quartiles than other parameters. Additionally, the AUROCs of TyG-WC and FLI were greater than the other parameters, with no significant difference when compared to each other. In particular, TyG-WC was shown to be a better predictor of NAFLD in relatively healthy individuals who are not obese or without metabolic syndrome or type 2 diabetes. The gold standard for diagnosis for NAFLD is liver biopsy. However, since performing such an invasive procedure on a large number of people is unreasonable, serum biomarkers and abdominal sonographies are usually used for diagnosis. Many attempts were targeted at finding an appropriate marker for NAFLD, one of which was FLI [12]. FLI involves measurements included in routine lab chemistries, as well as easily obtainable BMI and WC. In addition, FLI was associated with a high AUROC of 0.86 in a Korean population study [3]; this result suggests that FLI is a promising diagnostic tool for NAFLD. However, despite the presence of online tools that aid FLI calculation, the complicated formula hinders its widespread use in clinical practice. Recent studies have shown that the TyG index, a marker initially developed as an alternative index for insulin resistance, could be used as a diagnostic tool for NAFLD instead of FLI [7]. The TyG index has a simpler formula than FLI and has been widely used in many recent studies, as well as in clinical practice [19,20,21].

However, Khamseh et al. suggested that the TyG index with the addition of indices of obesity such as BMI and WC may be more accurate than the TyG index alone [14]. TyG-WC and TyG-BMI are also simply calculated by multiplying the TyG index by WC and BMI, respectively. The goal of their study was to evaluate the TyG-index, TyG-WC, and TyG-BMI for their prediction of NAFLD and liver fibrosis in overweight/obese individuals. The AUROCs in their study were 0.676 for thre TyG index, 0.675 for TyG-BMI, and 0.693 for TyG-WC, all of which are lower than the AUROCs produced in our study. The difference may have been due to the fact that our study was conducted with a large study sample; their study had only 184 overweight/obese participants. Additionally, we were able to compare the values of the different parameters with the participants’ abdominal sonography readings. We were able to find that higher values of all indices related to higher grades of fatty liver (Figure 2).

There are several possible explanations for our findings that TyG-BMI and TyG-WC were better predictors than TyG index alone. Accounting for the body composition may be essential in diseases related to metabolic syndrome, including NAFLD, which is metabolic syndrome’s hepatic manifestation. More specifically, our study showed that TyG-WC was superior to TyG-BMI in predicting NAFLD. WC is relatively more representative of visceral fat deposition, and BMI is considered a more general obesity marker that takes subcutaneous fat deposition into account [22]. The distribution of fat is more important than the total fat mass. Increased visceral fat in the abdomen is more related to insulin resistance and metabolic dysfunction, including hepatic steatosis, than subcutaneous fat [23,24,25]. This may explain our study results.

This study has several limitations. First, this was a single center study with a single ethnic cohort of Korean adults. Second, there is potential for selection bias, as study participants were self-referred for health screenings. Third, this study had a retrospective cross-sectional design. Future studies should include heterogeneous populations to ensure generalizability to other ethnicities. In addition, multi-centered longitudinal cohort studies should be conducted to determine whether high values in these parameters predict future occurrences of NAFLD. Another limitation that requires consideration is the fact that the diagnostic values of the parameters were compared with NAFLD found in liver ultrasonography, rather than other definitive diagnostic methods. Despite these potential limitations, our study is unique in that TyG-WC showed a similar predictability for NAFLD as the FLI, a specific diagnostic marker for NAFLD. To the best of our knowledge, this is the first study to compare the predictability for NAFLD between modified TyG indices and the FLI.

In conclusion, this study showed that TyG-WC can be a useful screening tool for NAFLD in Korean adults. TyG-WC has the advantages of being easily obtained and calculated with higher predictability compared to the FLI, especially in non-obese individuals without metabolic diseases.

## Figures and Tables

**Table 1:** Characteristics of the study population.

	NAFLD (+)	NAFLD (−)	*p*-Value
N	6856	10,721	
Male	4127 (60.2%)	3542 (33.0%)	<0.001
Age (years)	51.1 ± 11.8	47.5 ± 13.2	<0.001
BMI (kg/m^2^)	26.0 ± 3.6	22.4 ± 3.0	<0.001
Waist circumference (cm)	87.5 ± 9.9	76.3 ± 9.2	0.007
Fasting glucose (mg/dL)	105.4 ± 23.5	94.9 ± 14.8	<0.001
AST (IU/L)	31.1 ± 18.6	26.9 ± 16.5	<0.001
ALT (IU/L)	34.5 ± 25.1	21.7 ± 16.2	<0.001
Alkaline phosphatase (IU/L)	78.0 ± 22.6	70.0 ± 25.6	0.188
GGT (IU/L)	35.1 ± 33.2	21.4 ± 29.7	<0.001
Total cholesterol (mg/dL)	207.9 ± 14.2	202.0 ± 36.5	<0.001
HDL-cholesterol (mg/dL)	51.9 ± 11.5	61.1 ± 13.4	<0.001
LDL-cholesterol (mg/dL)	134.5 ± 33.0	124.8 ± 29.3	<0.001
Triglycerides (mg/dL)	152.8 ± 74.7	99.6 ± 49.7	<0.001
Current smoker (%)	1172 (18.1%)	994 (9.8%)	<0.001
Current exercise (%)	4373 (66.4%)	3041 (70.7%)	<0.001
Hypertension (%)	1936 (28.2%)	1543 (14.4%)	<0.001
Type 2 mellitus (%)	947 (13.8%)	426 (4.0%)	<0.001
Metabolic syndrome (%)	1966 (28.7%)	647 (6.0%)	<0.001

Values are presented as the mean ± SD or number (percentage). BMI, body mass index; AST, aspartate aminotransferase; ALT, alanine aminotransferase; GGT, γ-glutamyl transferase.

**Table 2:** Odds ratios for NAFLD according to quartiles of each parameter.

		All Subjects			
		Q1	Q2	Q3	Q4
TyG	All subjects				
	Unadjusted	1	2.712 (2.424–3.033)	6.198 (5.563–6.906)	17.056 (15.262–19.062)
	Adjusted	1	2.254 (1.996–2.545)	4.079 (3.620–4.597)	8.656 (7.633–9.817)
	Men				
	Unadjusted	1	2.308 (2.018–2.639)	4.375 (3.818–5.013)	9.013 (7.783–10.436)
	Adjusted	1	1.973 (1.709–2.278)	3.348 (2.889–3.880)	5.602 (4.761–6.592)
	Women				
	Unadjusted	1	2.669 (2.213–3.218)	5.751 (4.819–6.862)	17.151 (14.417–20.404)
	Adjusted	1	2.318 (1.895–2.835)	4.183 (3.448–5.075)	9.381 (7.722–11.396)
TyG-BMI	All subjects				
	Unadjusted	1	4.826 (4.192–5.558)	15.044 (13.126–17.243)	48.382 (42.014–55.716)
	Adjusted	1	4.180 (3.592–4.864)	10.842 (9.330–12.599)	26.815 (22.884–31.422)
	Men				
	Unadjusted	1	3.111 (2.701–3.583)	6.810 (5.897–7.864)	17.364 (14.786–20.393)
	Adjusted	1	2.700 (2.326–3.133)	5.155 (4.422–6.009)	10.435 (8.752–12.442)
	Women				
	Unadjusted	1	2.731 (2.168–3.442)	9.750 (7.890–12.050)	39.916 (32.339–49.266)
	Adjusted	1	2.438 (1.900–3.128)	7.846 (6.225–9.889)	25.194 (19.902–31.893)
TyG-WC	All subjects	1			
	Unadjusted	1	5.205 (4.454–6.084)	16.300 (14.014–18.958)	53.112 (45.436–62.084)
	Adjusted	1	4.913 (4.148–5.819)	13.565 (11.433–16.094)	34.515 (28.716–41.485)
	Men	1			
	Unadjusted	1	3.422 (2.933–3.993)	7.826 (6.688–9.157)	20.167 (16.918–24.040)
	Adjusted	1	3.059 (2.593–3.609)	6.238 (5.252–7.409)	12.912 (10.592–15.740)
	Women	1			
	Unadjusted	1	2.862 (2.210–3.706)	10.424 (8.223–13.213)	45.156 (35.672–57.161)
	Adjusted	1	2.672 (2.021–3.533)	9.275 (7.146–12.038)	32.163 (24.546–42.143)
FLI	All subjects	1			
	Unadjusted	1	4.598 (3.941–5.364)	15.490 (13.350–17.973)	51.042 (43.814–59.463)
	Adjusted	1	4.413 (3.731–5.220)	13.153 (11.109–15.572)	34.564 (28.831–41.437)
	Men	1			
	Unadjusted	1	3.369 (2.880–3.942)	8.544 (7.318–9.975)	20.628 (17.242–24.680)
	Adjusted	1	2.958 (2.503–3.495)	6.686 (5.649–7.914)	12.299 (10.018–15.099)
	Women	1			
	Unadjusted	1	2.643 (2.055–3.400)	9.373 (7.451–11.791)	43.152 (34.334–54.236)
	Adjusted	1	2.530 (1.929–3.320)	8.244 (6.389–10.638)	31.367 (24.054–40.903)

Adjusted for age, AST, ALT, SBP, DBP, smoking, exercise, and type 2 diabetes.

**Table 3:** Areas under the ROC curves for each parameter for predicting NAFLD.

Parameters	AUROC (95% CI)	Overall *p*-Value	Post Hoc *p*-Value				
All subjects							
BMI	0.790 (0.783–0.797)	<0.0001	Ref.				
WC	0.803 (0.796–0.810)		<0.0001	Ref.			
TyG index	0.773 (0.765–0.780)		<0.0001	<0.0001	Ref.		
TyG-BMI	0.827 (0.821–0.834)		<0.0001	<0.0001	<0.0001	Ref.	
TyG-WC	0.832 (0.826–0.839)		<0.0001	<0.0001	<0.0001	0.0020	Ref.
FLI	0.835 (0.828–0.841)		<0.0001	<0.0001	<0.0001	<0.0001	0.132
Male							
BMI	0.730 (0.718–0.742)		Ref.				
WC	0.742 (0.731–0.754)		<0.0001	Ref.			
TyG index	0.724 (0.711–0.736)	<0.0001	<0.0001	<0.0001	Ref.		
TyG-BMI	0.774 (0.763–0.786)		<0.0001	<0.0001	<0.0001	Ref.	
TyG-WC	0.783 (0.772–0.794)		<0.0001	<0.0001	<0.0001	0.0014	Ref.
FLI	0.783 (0.772–0.794)		<0.0001	<0.0001	<0.0001	0.0001	0.893
Female							
BMI	0.794 (0.784–0.805)		Ref.				
WC	0.808 (0.798–0.818)		0.0008	Ref.			
TyG index	0.772 (0.761–0.784)	<0.0001	0.0040	<0.0001	Ref.		
TyG-BMI	0.832 (0.822–0.841)		<0.0001	<0.0001	<0.0001	Ref.	
TyG-WC	0.840 (0.831–0.850)		<0.0001	<0.0001	<0.0001	0.0036	Ref.
FLI	0.839 (0.830–0.849)		<0.0001	<0.0001	<0.0001	0.0053	0.5569

AUROC, area under the ROC curve; BMI, body mass index; WC, waist circumference; TyG, triglyceride and glucose; FLI, fatty liver index.

**Table 4:** Areas under the ROC curves for each parameter for predicting NAFLD according to metabolic status.

**Non-Obese**			**Obese**		
**Parameters**	**AUROC (95% CI)**	**Overall** ***p*-value**	**Parameters**	**AUROC (95% CI)**	**Overall** ***p*-value**
BMI	0.736 (0.725–0.747)	<0.0001	BMI	0.637 (0.621–0.653)	<0.0001
WC	0.762 (0.752–0.772)		WC	0.660 (0.645–0.676)	
TyG index	0.759 (0.748–0.770)		TyG index	0.688 (0.673–0.704)	
TyG-BMI	0.799 (0.789–0.808)		TyG-BMI	0.710 (0.695–0.725)	
TyG-WC	0.804 (0.795–0.814)		TyG-WC	0.716 (0.701–0.731)	
FLI	0.804 (0.795–0.814)		FLI	0.725 (0.711–0.740)	
**Without DM**			**With DM**		
**Parameters**	**AUROC (95% CI)**	**Overall** ***p*-value**	**Parameters**	**AUROC (95% CI)**	**Overall** ***p*-value**
BMI	0.790 (0.783–0.798)	<0.0001	BMI	0.702 (0.669–0.734)	<0.0001
WC	0.802(0.795–0.810)		WC	0.687 (0.654–0.720)	
TyG index	0.765(0.757–0.773)		TyG index	0.672 (0.638–0.706)	
TyG-BMI	0.826 (0.819–0.833)		TyG-BMI	0.727 (0.695–0.759)	
TyG-WC	0.831 (0.824–0.838)		TyG-WC	0.716 (0.683–0.748)	
FLI	0.834 (0.827–0.841)		FLI	0.733 (0.701–0.765)	
**Without metabolic syndrome**			**With Metabolic syndrome**		
**Parameters**	**AUROC (95% CI)**	**Overall** ***p*-value**	**Parameters**	**AUROC (95% CI)**	**Overall** ***p*-value**
BMI	0.760 (0.752–0.769)	<0.0001	BMI	0.621 (0.597–0.646)	<0.0001
WC	0.777 (0.769–0.786)		WC	0.626 (0.601–0.651)	
TyG index	0.740 (0.731–0.749)		TyG index	0.647 (0.622–0.671)	
TyG-BMI	0.805 (0.797–0.813)		TyG-BMI	0.665 (0.641–0.689)	
TyG-WC	0.812 (0.804–0.820)		TyG-WC	0.669 (0.644–0.693)	
FLI	0.814 (0.806–0.822)		FLI	0.687 (0.663–0.711)	

## Data Availability

The datasets generated during and/or analyzed during the current study are available from the corresponding author on reasonable request.
